# Vitamin C—Beyond Deficiency: Mechanisms, Clinical Applications, Formulation and Dosing Considerations, and Safety Across Stress-Responsive Conditions

**DOI:** 10.3390/nu18142319

**Published:** 2026-07-15

**Authors:** Yonghyun Yoon, Jihyo Hwang, Chan-Mo Yang, Seungbeom Kim, Jonghyeok Lee, Jong-Jin Lee, Myunghoon Moon, King Hei Stanley Lam

**Affiliations:** 1 Department of Orthopaedic Surgery, Gangnam Sacred Heart Hospital, Hallym University College of Medicine, 1 Singil-ro, Yeongdeungpo-gu, Seoul 07441, Republic of Korea; mgyyh00@gmail.com (Y.Y.);; 2 Incheon Terminal Orthopedic Surgery Clinic, Inha-ro 489 beon-gil, Namdong-gu, Incheon 21574, Republic of Korea; 3 International Academy of Regenerative Medicine, Inha-ro 489 beon-gil, Namdong-gu, Incheon 21574, Republic of Korea; 4 Department of Psychiatry, Wonkwang University Hospital, Wonkwang University School of Medicine, Iksan 54538, Republic of Korea; 5 Miso Pain Clinic, 1569, Bongyeong-ro, Yeongtong-gu, Suwon-si 16703, Republic of Korea; 6 Bareun Neurosurgery Clinic, 39, Daenong-ro, Heungdeok-gu, Cheongju-si 28402, Republic of Korea; 7 Ceramique Clinic, Seoul 06123, Republic of Korea; 8 Naeuram Clinic, Seoul 06611, Republic of Korea; 9 The Department of Clinical Research, International Academy of Musculoskeletal Medicine, Hong Kong, China; 10 Faculty of Medicine, The University of Hong Kong, Hong Kong, China; 11 Faculty of Medicine, The Chinese University of Hong Kong, Hong Kong, China

**Keywords:** vitamin C, ascorbic acid, clinical nutrition, oxidative stress, inflammation, tissue repair, personalized nutrition, intravenous ascorbate, formulation, safety

## Abstract

Vitamin C (L-ascorbic acid) is an essential micronutrient involved in collagen biosynthesis, redox regulation, immune function, endothelial biology, carnitine synthesis, neurotransmitter metabolism, and non-heme iron absorption. Dietary reference values are designed primarily to prevent deficiency in general populations, but vulnerability to low-vitamin C status may increase during trauma, surgery, chronic inflammation, malignancy, metabolic disease, smoking, poor intake, environmental exposure, and tissue repair. This narrative review synthesizes mechanistic, pharmacokinetic, clinical, and safety evidence on vitamin C as a stress-responsive micronutrient. Evidence is reviewed across tissue repair and wound healing, orthopedic recovery and selected complex regional pain syndrome risk contexts, fatigue, neuropsychiatric vulnerability, cancer-supportive care, vascular homeostasis, dermatologic biology, and preliminary microbiota–gut–brain axis hypotheses. The strength of evidence differs substantially across domains: biochemical functions and deficiency correction are well established, whereas benefits of supraphysiologic oral supplementation in vitamin C-replete patients remain uncertain. Oral nutritional supplementation is distinguished from intravenous pharmacologic ascorbate, with attention to route, formulation, dose division, gastrointestinal tolerance-limited adjustment, and safety monitoring. Because evidence for high-dose oral supplementation remains limited and condition-specific, such use should be individualized, time-limited, and clinician-monitored rather than presented as a population-level recommendation or evidence-defined therapeutic target. Taken together, the clinical value of vitamin C depends on baseline status, patient vulnerability, route, formulation, dosing interval, clinical endpoint, and safety review.

## 1. Introduction

Vitamin C is a familiar micronutrient, but its clinical interpretation continues to span deficiency-prevention nutrition, individualized supplementation, and pharmacologic ascorbate use. Conventional dietary guidance focuses on preventing scurvy and meeting population reference intakes, whereas higher-dose oral or intravenous approaches have been explored in selected contexts such as infection, inflammation, fatigue, cancer-related supportive care, pain, wound healing, and recovery from physiological stress [1]. This review distinguishes established biochemical functions and deficiency correction from association-based interpretations, pharmacologic interventions, and claims that remain unsupported. Accordingly, evidence is interpreted by distinguishing established biochemical function, associations with low-vitamin C status, benefits derived from correcting deficiency or insufficiency, and evidence for supraphysiologic supplementation in vitamin C-replete individuals.

Humans cannot synthesize vitamin C endogenously because of the evolutionary loss of L-gulonolactone oxidase activity and must depend on dietary or supplemental intake [1,2]. In humans, vitamin C status is determined by intake, intestinal absorption, renal conservation and excretion, tissue uptake, oxidative consumption, and disease-related demand. Consequently, a single population-level intake target may not fully describe the clinical context of patients exposed to smoking, infection, trauma, surgery, chronic inflammation, metabolic disease, environmental pollutants, pharmacologic oxidative stress, malignancy, or tissue-repair demands, although this does not by itself establish a need for supraphysiologic supplementation [1,2].

The clinical relevance of vitamin C is grounded in several biological roles. Vitamin C is an essential cofactor for prolyl and lysyl hydroxylases, supporting collagen hydroxylation and extracellular matrix integrity [3]. It also functions as an electron donor and redox buffer, participates in antioxidant recycling, supports immune-cell function, contributes to endothelial nitric oxide bioavailability, and interacts with redox-sensitive inflammatory pathways [2]. Vitamin C is also increasingly recognized as a cofactor for selected Fe(II)- and α-ketoglutarate-dependent dioxygenases involved in oxygen sensing and epigenetic regulation, including hypoxia-inducible factor hydroxylation and DNA or histone demethylation pathways [4,5]. These mechanisms expand the biological rationale for context-dependent assessment but do not establish clinical benefit from supraphysiologic supplementation in vitamin C-replete patients. In addition, vitamin C is involved in carnitine synthesis and neurotransmitter metabolism, providing mechanistic plausibility—but not sufficient clinical proof—for its relevance to fatigue, mood symptoms, cognitive function, and neuropsychiatric vulnerability in nutritionally vulnerable patients [6].

A useful clinical frame avoids two extremes: vitamin C is neither a simple dietary checkbox nor a universal disease-directed therapy. It may be better understood as a demand-sensitive cofactor whose clinical relevance may increase when tissue repair, oxidative stress, inflammation, or poor intake raises physiologic requirements [7,8]. This interpretation may be particularly useful in orthopedic recovery, neuropsychiatric vulnerability, and cancer-supportive care, where symptom burden and nutritional risk often coexist, while recognizing that evidence strength differs by clinical endpoint.

Clinically, vitamin C use depends on route, formulation, dose unit, dosing interval, baseline status, gastrointestinal tolerance, renal risk, iron metabolism, hydration, and oncologic context. Oral vitamin C is most relevant to nutritional repletion, redox support, collagen turnover, daily supplementation, and, in selected monitored contexts, gastrointestinal tolerance-limited dose adjustment rather than validated requirement-based titration. Intravenous vitamin C is a distinct pharmacologic intervention that bypasses intestinal transport limitations and requires appropriate medical supervision [7,8].

**Figure 1:**
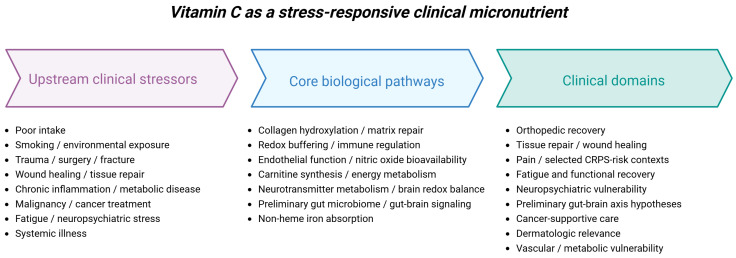
Conceptual framework of vitamin C as a stress-responsive clinical micronutrient. Abbreviations: CRPS, complex regional pain syndrome.

The overall conceptual framework of vitamin C as a stress-responsive clinical micronutrient, with evidence interpreted according to clinical context and route of administration, is summarized in Figure 1.

This review summarizes how vitamin C biology may inform clinical decision points for orthopedic injury, pain, tissue-repair demand, fatigue, neuropsychiatric symptoms, cancer-related supportive care, and increased oxidative or inflammatory burden. Practical considerations are organized around patient selection, route, formulation, divided dosing, gastrointestinal tolerance-limited adjustment, and safety review.

Previous reviews have addressed vitamin C physiology, immune function, dermatologic biology, cancer-related intravenous ascorbate, or selected musculoskeletal outcomes [2,3,9,10], but fewer have integrated route-specific exposure, formulation considerations, patient-selection logic, safety boundaries, and evidence-sensitive interpretation across stress-responsive clinical contexts. The added value of this review is to synthesize vitamin C as a context-dependent clinical micronutrient while separating nutritional repletion, maintenance supplementation, selected monitored oral supplementation above conventional ranges, and intravenous pharmacologic ascorbate.

Upstream clinical stressors, including poor intake, smoking or environmental exposure, trauma, surgery, fracture, wound-healing demand, chronic inflammation, metabolic disease, malignancy, cancer treatment, fatigue, neuropsychiatric stress, and systemic illness, may increase vitamin C turnover or vulnerability to low-vitamin C status. These stressors intersect with core biological pathways involving collagen hydroxylation and matrix repair, redox buffering, immune regulation, endothelial function, nitric oxide bioavailability, carnitine synthesis, energy metabolism, neurotransmitter metabolism, brain redox balance, preliminary gut microbiome–gut–brain signaling hypotheses, and non-heme iron absorption. These pathways provide a rationale for evaluating vitamin C status across orthopedic recovery, tissue repair, wound healing, pain and selected complex regional pain syndrome risk contexts, fatigue, neuropsychiatric vulnerability, preliminary gut–brain axis hypotheses, cancer-supportive care, dermatologic relevance, and vascular or metabolic vulnerability.

## 2. Methods

This narrative review synthesizes evidence on vitamin C across biological, pharmacokinetic, nutritional, clinical, and safety domains. The aim was to develop a clinically oriented synthesis of vitamin C as a stress-responsive micronutrient; the review was not designed as a disease-specific guideline, formal systematic review, or quantitative meta-analysis [11,12].

The literature search focused on vitamin C physiology, collagen biosynthesis, redox regulation, immune function, endothelial biology, carnitine synthesis, neurotransmitter metabolism, oral and intravenous pharmacokinetics, complex regional pain syndrome risk reduction, musculoskeletal recovery, wound healing, cancer-supportive care, fatigue, skin biology, neuropsychiatric symptoms, nephrolithiasis, renal safety, iron metabolism, and formulation-dependent considerations.

Relevant literature was identified through searches of PubMed, Scopus, Web of Science, Google Scholar, official dietary reference documents, and selected historically influential papers on high-dose oral vitamin C and gastrointestinal tolerance-limited dosing concepts.

Searches covered available records through June 2026, with additional citation tracking from key reviews, pharmacokinetic studies, clinical guidelines, dietary reference documents, and historically influential clinical reports. Search terms included “vitamin C,” “ascorbic acid,” “clinical nutrition,” “oxidative stress,” “collagen synthesis,” “wound healing,” “fracture healing,” “tendon healing,” “complex regional pain syndrome,” “orthopedic surgery,” “pain,” “cancer fatigue,” “intravenous vitamin C,” “oral vitamin C,” “gastrointestinal tolerance,” “bowel tolerance,” “Cathcart,” “liposomal vitamin C,” “bioavailability,” “nephrolithiasis,” “oxalate,” “kidney stones,” “depression,” “neuropsychiatric symptoms,” “fatigue,” “skin health,” “endothelial function,” “smoking,” “environmental pollution,” and “heavy metals.” Terms were combined using Boolean operators according to each topic, for example (“vitamin C” OR “ascorbic acid”) AND (“wound healing” OR “fracture healing” OR “complex regional pain syndrome” OR “fatigue” OR “cancer” OR “intravenous vitamin C” OR “bioavailability” OR “nephrolithiasis”). English-language human studies, systematic reviews, meta-analyses, randomized trials, pharmacokinetic studies, clinical guidelines, dietary reference documents, and clinically relevant mechanistic studies were prioritized.

Within each clinical domain, priority was given to higher-level clinical evidence when available, including randomized trials, systematic reviews, meta-analyses, clinical guidelines, pharmacokinetic studies, and dietary reference documents. Preclinical and biochemical studies were included when they provided a mechanistic basis for clinical interpretation, particularly in relation to collagen synthesis, redox balance, immune function, tissue repair, fatigue, and neuropsychiatric relevance. Historical orthomolecular literature was included selectively when it influenced contemporary clinical practice or dosing concepts, but it was interpreted as historical or hypothesis-generating rather than as guideline-level evidence.

Because this was a narrative review, no formal risk-of-bias assessment, evidence grading, PRISMA flow diagram, or meta-analysis was performed. Evidence was interpreted according to clinical domain, route of administration, formulation, dose, safety profile, patient context, baseline vitamin C status when reported, and whether the evidence supported biochemical function, deficiency correction, association, or supraphysiologic supplementation.

## 3. Biological Rationale for Clinical Use

Although vitamin C is often introduced as a water-soluble antioxidant, this description is too narrow for clinical interpretation [2,6]. It also acts as an electron donor, enzyme cofactor, redox buffer, immune-cell nutrient, collagen-related cofactor, and regulator of pathways involved in tissue repair, fatigue, vascular integrity, and neuropsychiatric function. These roles may become more clinically relevant when oxidative, inflammatory, metabolic, pharmacologic, oncologic, or repair-related stress increases biological demand.

### 3.1. Collagen, Extracellular Matrix, and Tissue Integrity

One of the most clinically established functions of vitamin C is its role as a cofactor for prolyl and lysyl hydroxylases, enzymes required for the post-translational hydroxylation of collagen [3,13]. Hydroxylation of proline and lysine residues is essential for collagen triple-helix stability, cross-linking, and extracellular matrix integrity. Severe vitamin C deficiency produces scurvy, characterized by impaired wound healing, capillary fragility, gingival bleeding, perifollicular hemorrhage, musculoskeletal pain, and connective tissue weakness. Biochemically, vitamin C deficiency is commonly defined using low plasma ascorbate concentrations, with severe deficiency often considered at approximately <11 µmol/L, although thresholds vary by assay, compartment, and clinical context. Clinical scurvy generally reflects sustained depletion over weeks to months rather than short-term fluctuation, and leukocyte vitamin C may better reflect tissue stores but is less widely available in routine practice [1,14].

In contemporary practice, overt scurvy is less common in many settings, yet marginal vitamin C status may be clinically relevant when repair demand is high [13]. Orthopedic injury, fracture healing, tendon or ligament injury, postoperative recovery, pressure injury, chronic wounds, smoking, diabetes, advanced age, frailty, poor protein intake, and chronic inflammation represent settings in which collagen-related nutrient adequacy may deserve clinical attention [9,15]. From a musculoskeletal perspective, vitamin C is a necessary cofactor for collagen-dependent repair biology. It cannot replace fixation, rehabilitation, biologic procedures, or wound care, but an inadequate status may compromise the biological conditions required for collagen maturation and tissue recovery [9].

This mechanism is particularly relevant to the biological rationale for orthopedic and regenerative medicine, where fracture, tendon injury, ligament injury, fasciopathy, enthesopathy, surgery, and wound complications require coordinated extracellular matrix remodeling. Vitamin C contributes to collagen maturation, fibroblast activity, angiogenesis, epithelial repair, and matrix stability; its role is permissive and cofactor-based without implying independent regenerative efficacy [9,16].

### 3.2. Redox Regulation, Inflammation, and Immune Demand

Vitamin C functions as a major water-soluble electron donor and participates in redox buffering within plasma, immune cells, endothelial cells, and other tissues [2,17]. It can directly neutralize reactive oxygen and nitrogen species and can also regenerate other antioxidants, including vitamin E, from their oxidized forms. This role is particularly relevant during physiological stress, when oxidative burden and inflammatory signaling increase.

Immune cells accumulate vitamin C at concentrations higher than those found in plasma [2]. Neutrophils, monocytes, and lymphocytes require redox protection during activation, migration, phagocytosis, and oxidative burst activity. During infection, trauma, surgery, systemic inflammation, or malignancy, vitamin C turnover may increase as immune and inflammatory processes consume available antioxidant capacity. This may create a functional state in which routine intake is sufficient to prevent classical deficiency but may not fully address increased biological demand.

Clinically, this redox and immune-cell biology supports assessment of vitamin C status in patients with acute injury, postoperative stress, chronic inflammatory disease, metabolic syndrome, diabetes, obesity, smoking exposure, environmental pollutant exposure, recurrent infection, or cancer-related systemic stress. In these settings, adequate vitamin C status may contribute to immune, endothelial, and connective-tissue resilience.

### 3.3. Endothelial Function and Vascular Homeostasis

At the vascular level, vitamin C helps preserve endothelial function by protecting nitric oxide from oxidative inactivation, maintaining redox balance, and supporting collagen integrity in the vessel wall [18,19]. This provides a plausible mechanistic explanation for the association between low-vitamin C status and impaired vascular function in high-oxidative-stress states.

Clinically, adequate vitamin C status may contribute to endothelial and vascular redox homeostasis, particularly in patients with low intake or increased oxidative burden. Smokers, patients with metabolic syndrome, diabetes, obesity, chronic inflammation, environmental pollutant exposure, or poor diet quality may have greater vulnerability to low-vitamin C status than metabolically healthy individuals with adequate intake.

This vascular perspective also has implications beyond cardiology. Microvascular dysfunction, oxidative stress, and endothelial activation contribute to tissue repair, postoperative recovery, neuropathic pain, wound healing, and cancer-related systemic illness. Therefore, vascular homeostasis provides one mechanistic bridge between vitamin C biology and several clinical domains, without establishing a disease-specific treatment effect.

### 3.4. Carnitine Synthesis, Mitochondrial Function, and Fatigue

Vitamin C is required for the activity of enzymes involved in carnitine biosynthesis [20]. Carnitine is necessary for the transport of long-chain fatty acids into mitochondria, where they undergo beta-oxidation and contribute to ATP generation. This pathway provides a biologically plausible connection between low-vitamin C status and fatigue, lethargy, weakness, and reduced physical resilience.

Fatigue is a nonspecific symptom and should not be attributed to vitamin C status alone. Nevertheless, vitamin C insufficiency may contribute to fatigue in selected patients when poor intake, inflammation, malignancy, chronic illness, oxidative stress, or increased metabolic demand is present. Cancer-related fatigue, post-illness fatigue, frailty, chronic inflammatory disease, and postoperative recovery are clinical contexts in which vitamin C status may deserve attention as part of a broader nutritional assessment.

Fatigue requires broad evaluation, including anemia, thyroid dysfunction, sleep disturbance, depression, medication effects, systemic inflammation, sarcopenia, malnutrition, malignancy, and treatment-related factors. Vitamin C status becomes relevant when fatigue is accompanied by poor intake, inflammation, chronic illness, malignancy, or increased oxidative stress.

### 3.5. Neurotransmitter Metabolism and Neuropsychiatric Relevance

Vitamin C is highly concentrated in the central nervous system and is involved in several pathways that may be relevant to neuropsychiatric function [21]. It participates in catecholamine metabolism, supports antioxidant defense in neural tissue, and may influence neuroinflammatory and redox-sensitive signaling. Vitamin C is also involved in the recycling of cofactors required for monoamine synthesis and in the regulation of oxidative stress within the brain.

Low-vitamin C status has been associated with irritability, low mood, fatigue, cognitive complaints, and depressive symptoms [22]. These findings are most applicable to psychiatric and psychosomatic practice when restricted diets, chronic stress, smoking, alcohol use, systemic inflammation, cancer, chronic pain, or poor overall nutritional status are present. They do not establish vitamin C as a primary psychiatric treatment; depression, anxiety, cognitive impairment, and fatigue still require standard diagnostic evaluation and evidence-based care.

In neuropsychiatric practice, vitamin C status is a modifiable nutritional variable in patients with deficiency, poor diet quality, oxidative stress, chronic pain, cancer, smoking, alcohol use, or systemic illness. Its role is physiologic and supportive, centered on brain redox balance and neurotransmitter-related pathways within standard psychiatric and medical care.

### 3.6. Iron Metabolism and Clinical Caution

Vitamin C enhances the absorption of non-heme iron by reducing ferric iron to the more absorbable ferrous form [23,24]. This effect may be beneficial in patients with low dietary iron intake or iron deficiency, particularly when plant-based non-heme iron is the predominant source. However, this same property requires caution in patients with iron overload, hereditary hemochromatosis, repeated transfusion exposure, or selected oncologic and hematologic conditions.

Thus, the same biochemical property can be beneficial in one patient and undesirable in another. For patients at risk for iron overload, prolonged high-dose vitamin C supplementation should be guided by iron status rather than assumed to be universally benign [25]. Ferritin, transferrin saturation, and hematologic context are relevant when high-dose supplementation is planned.

### 3.7. Integrative Clinical Interpretation

The mechanisms reviewed above link vitamin C to collagen synthesis, extracellular matrix stability, redox regulation, immune function, endothelial biology, carnitine synthesis, neurotransmitter metabolism, and iron handling. These pathways provide mechanistic rationale for the clinical domains discussed below, but mechanistic plausibility alone is insufficient to support disease-directed claims or supraphysiologic supplementation in vitamin C-replete patients.

## 4. Clinical Domains of Vitamin C Relevance

Vitamin C has been investigated across diverse clinical settings, but its interpretation should remain domain-specific and evidence-sensitive. Its clinical relevance is most defensible when increased oxidative stress, inflammation, tissue injury, poor intake, malignancy, pharmacologic burden, or repair demand overlaps with low-vitamin C status or pathways dependent on collagen biology, redox balance, immune function, or endothelial homeostasis.

The following domains are reviewed from a clinical nutrition perspective: orthopedic injury and postoperative recovery, pain and selected complex regional pain syndrome (CRPS) risk contexts, wound healing and musculoskeletal repair, cancer-supportive care, fatigue, neuropsychiatric symptoms, skin biology, vascular homeostasis, and environmental or metabolic stress.

### 4.1. Orthopedic Injury, Pain, and Complex Regional Pain Syndrome Risk Reduction

Orthopedic injury may represent a high-demand setting because trauma, fracture, surgery, inflammation, immobilization, and tissue repair often converge in the same patient [13]. Vitamin C supports collagen hydroxylation, extracellular matrix maturation, endothelial redox balance, immune response, and oxidative-stress regulation. These mechanisms provide a biological rationale for considering vitamin C status in fracture healing, postoperative recovery, wound healing, tendon and ligament biology, and post-traumatic pain.

The most clinically studied orthopedic application of vitamin C is the reduction of complex regional pain syndrome type I risk after fracture or extremity surgery [26,27,28]. Several randomized trials and meta-analyses have evaluated oral vitamin C, commonly at 500 mg/day for approximately 45–50 days, after distal radius fracture or orthopedic trauma. Some studies have suggested a reduction in CRPS-I incidence, whereas others have reported less consistent effects. Current evidence supports cautious consideration of vitamin C in selected post-traumatic or postoperative settings where CRPS risk is clinically relevant, while recognizing that trial results are not fully consistent.

The rationale for vitamin C in CRPS risk reduction is biologically plausible but not definitive. CRPS involves complex interactions among neurogenic inflammation, oxidative stress, endothelial dysfunction, microvascular dysregulation, peripheral and central sensitization, and abnormal tissue response after injury [29,30]. Vitamin C intersects with several of these pathways through antioxidant defense, endothelial function, collagen repair, and inflammatory regulation, but these mechanisms do not establish a generalized analgesic effect. However, CRPS is multifactorial, and vitamin C supplementation cannot replace early diagnosis, pain control, edema management, mobilization, rehabilitation, desensitization therapy, and appropriate multidisciplinary care.

Beyond CRPS, the rationale remains mechanistic and is most relevant to pain states linked to tissue injury, oxidative burden, microvascular dysfunction, or impaired repair. In orthopedic practice, these contexts include fracture, postoperative wounds, tendon or ligament injury, delayed soft-tissue recovery, smoking exposure, diabetes, frailty, poor diet quality, and high inflammatory burden.

### 4.2. Regeneration, Wound Healing, and Musculoskeletal Recovery

In healing tissues, vitamin C is closely linked to tissue repair because collagen synthesis, fibroblast activity, extracellular matrix remodeling, angiogenesis, epithelial repair, and oxidative-stress control are relevant to wound healing and musculoskeletal recovery [3,13]. These mechanisms support attention to vitamin C status after surgery, trauma, chronic wounds, pressure injuries, tendon or ligament injury, muscle injury, fracture, and regenerative procedures, especially when dietary intake is low or repair demand is high.

In regenerative medicine, the most cautious interpretation is necessary but not sufficient: adequate vitamin C status may support biochemical conditions for repair, but vitamin C is not a regenerative therapy by itself. It cannot substitute for platelet-rich plasma, cell-based therapy, prolotherapy, extracorporeal shockwave therapy, surgery, immobilization, rehabilitation, or standard wound management.

When a tissue-repair intervention is technically successful, recovery can still be limited by poor nutrition, smoking, diabetes, sarcopenia, low protein intake, vitamin deficiency, chronic inflammation, or oxidative stress. Vitamin C should therefore be considered together with protein adequacy, B vitamins, vitamin D, magnesium, zinc, glycemic control, sleep, mechanical loading, and rehabilitation.

In wound healing, vitamin C deficiency is classically associated with impaired collagen formation, capillary fragility, bleeding tendency, and delayed tissue repair. Even in the absence of overt scurvy, patients with marginal intake or increased repair demand may be vulnerable to suboptimal vitamin C status. Older adults, institutionalized patients, smokers, alcohol users, patients with malabsorption, patients with restrictive diets, and patients with chronic inflammatory or malignant disease may require particular attention.

Clinical evidence for accelerated musculoskeletal healing remains heterogeneous, but maintaining adequate vitamin C status is reasonable when tissue repair demand is high or baseline intake is low [13], without implying reliable acceleration of radiographic or functional healing. Any high-dose plan, if considered, should be individualized and integrated with protein adequacy, metabolic control, rehabilitation, mechanical loading, sleep optimization, and smoking cessation.

### 4.3. Cancer-Supportive Care

Vitamin C has a long and controversial history in oncology, so route must be stated explicitly. Oral supplementation primarily addresses nutritional adequacy, redox homeostasis, collagen biology, immune support, and symptom-oriented supportive care. Intravenous ascorbate bypasses intestinal absorption limits and can achieve plasma concentrations that conventional oral dosing cannot.

Cancer patients may be vulnerable to vitamin C deficiency or insufficiency for several reasons, including poor appetite, reduced intake of fresh fruits and vegetables, nausea, vomiting, mucositis, systemic inflammation, cachexia, oxidative stress, infection, surgery, chemotherapy, radiation therapy, and advanced disease burden [31]. In this context, assessment of vitamin C status and nutritional adequacy may be clinically meaningful, especially in patients with poor intake, fatigue, poor wound healing, inflammation, frailty, or reduced quality of life.

Intravenous ascorbate has been evaluated in oncology supportive care and early-phase studies [10,32]. Proposed mechanisms include redox modulation and pharmacologic hydrogen peroxide generation at high plasma concentrations, whereas reported clinical outcomes have mainly involved symptom relief, treatment-related fatigue, and quality of life. Results remain heterogeneous, and definitive evidence for survival benefit remains lacking. Its role should therefore be described as investigational or supportive, not curative.

Patient communication should make this distinction explicit: oral supplementation is a nutritional intervention, whereas high-dose intravenous (IV) ascorbate is a monitored medical treatment with different pharmacokinetics, safety screening, and potential interactions.

In oncology support, vitamin C is most appropriately discussed in relation to nutritional insufficiency, fatigue, quality of life, wound healing, postoperative recovery, and treatment-related symptoms. During chemotherapy, radiation therapy, immunotherapy, or targeted therapy, use requires coordination with the treating oncologist because redox-active interventions may interact with regimen-specific mechanisms and timing [32].

In hematologic oncology and transplantation settings, recent pilot randomized evidence suggests that vitamin C supplementation can correct low plasma vitamin C levels; however, larger adequately powered trials are needed before conclusions can be drawn regarding complications, treatment tolerance, or survival-related outcomes [33].

### 4.4. Fatigue and Functional Recovery

Fatigue is one of the most common symptoms encountered in clinical practice and is especially prevalent in patients with cancer, chronic inflammation, infection, postoperative recovery, metabolic disease, chronic pain, depression, sleep disturbance, and frailty. Because fatigue is multifactorial, vitamin C status should be evaluated as one nutritional variable within a broader assessment that includes anemia, thyroid dysfunction, sleep disturbance, depression, anxiety, chronic infection, inflammatory disease, renal or hepatic dysfunction, medication effects, nutritional status, protein intake, sarcopenia, B-vitamin deficiency, vitamin D status, magnesium status, and cancer-related factors. Its roles in carnitine biosynthesis, redox balance, endothelial homeostasis, immune function, and catecholamine metabolism provide mechanistic plausibility for fatigue and functional recovery, particularly when fatigue coexists with poor intake, systemic inflammation, malignancy, postoperative recovery, or increased oxidative stress.

Clinical studies of vitamin C for fatigue have included intravenous administration in selected populations, including office workers, cancer patients, and post-viral or chronic fatigue contexts [34,35]. Some studies suggest symptomatic improvement in selected populations, but results are not uniform, and baseline vitamin C status is not always measured [34,35]. The heterogeneity of fatigue mechanisms makes it difficult to generalize findings across populations or to infer benefit in vitamin C-replete individuals [31,35].

### 4.5. Neuropsychiatric Symptoms and Brain Health

Vitamin C is highly concentrated in the central nervous system, where it participates in antioxidant defense, neurotransmitter-related pathways, and redox-sensitive neuronal function. The brain’s high oxygen use, lipid-rich structure, and active neurotransmitter metabolism make low-vitamin C status clinically relevant to consider when neuropsychiatric or cognitive symptoms coexist with nutritional vulnerability or systemic illness.

Vitamin C is involved in catecholamine metabolism and may plausibly influence pathways related to dopamine, norepinephrine, serotonin, glutamate, GABAergic signaling, neuroinflammation, and oxidative stress. Deficiency states have been associated with irritability, fatigue, low mood, depression, and cognitive complaints [22]. In some clinical studies, vitamin C supplementation has been associated with improvement in mood or psychological symptoms, particularly in individuals with low baseline status, institutionalized populations, or medically ill populations [36].

Depression, anxiety, cognitive decline, sleep disturbance, and fatigue require standard clinical evaluation and evidence-based management. Vitamin C status assessment is most justified when these symptoms coexist with restricted diets, low fruit and vegetable intake, smoking exposure, alcohol use, chronic stress, chronic pain, inflammatory disease, cancer, frailty, or systemic illness.

For psychiatrists and psychosomatic clinicians, the clinical value lies in recognizing overlapping contributors: mood symptoms, fatigue, chronic pain, cognitive complaints, poor sleep, low physical activity, inflammation, metabolic syndrome, smoking, medication burden, and suboptimal nutrition. Vitamin C status can be evaluated alongside sleep, exercise, diet quality, psychotherapy, pharmacotherapy, and treatment of systemic disease.

### 4.6. Preliminary Gut–Brain Axis, Microbiome Remodeling, and Neuroimmune Signaling Hypotheses

Recent microbiome studies have raised the hypothesis that vitamin C status or supplementation may interact with the microbiota–gut–brain axis in ways that could be relevant to neuropsychiatric vulnerability. This pathway is unlikely to depend solely on direct molecular delivery of vitamin C to the brain. A more cautious interpretation is that adequate or supplemental vitamin C may alter the intestinal redox and microbial environment, with potential downstream effects on microbial metabolites, epithelial barrier function, immune signaling, and neuroimmune communication [37,38].

Human pilot and randomized data suggest that vitamin C supplementation may alter gut microbial composition in selected populations. In healthy individuals, high-dose vitamin C supplementation has been associated with shifts in gut bacterial populations, and a recent randomized, double-blind, placebo-controlled trial in healthy young adults with suboptimal vitamin C status linked vitamin C supplementation to improved mental vitality through gut microbiota-related changes [39,40]. These findings support a preliminary microbiome-mediated hypothesis, although the available evidence remains early, population-specific, and insufficient to establish causal neuropsychiatric benefit.

Mechanistically, the microbiota–gut–brain axis communicates through immune, endocrine, neural, and metabolic routes, including microbial metabolites such as short-chain fatty acids (SCFAs), vagal signaling, intestinal barrier regulation, and microglia-related neuroimmune pathways. Short-chain fatty acids, especially acetate, propionate, and butyrate, may influence neuroinflammation, blood–brain barrier integrity, microglial activation, and neurotransmitter-related signaling [37,38,41]. In this context, vitamin C is more appropriately viewed as a potential nutritional modifier of the gut redox–microbial ecosystem than as a stand-alone psychiatric intervention.

This hypothesis may be particularly relevant when mood symptoms, fatigue, cognitive complaints, chronic stress, or pain coexist with poor diet quality, low fruit and vegetable intake, smoking, inflammation, metabolic disease, or suboptimal vitamin C status. However, current evidence does not support vitamin C as a primary treatment for psychiatric disease. Future studies should combine baseline vitamin C assessment, microbiome sequencing, metabolomics, short-chain-fatty-acid profiling, inflammatory markers, vagal or autonomic measures, and validated neuropsychiatric outcomes.

### 4.7. Skin Biology and Dermatologic Relevance

Vitamin C is concentrated in the skin and contributes to collagen synthesis, antioxidant protection, barrier function, wound repair, and photoprotection biology [3]. The skin is one of the organs in which vitamin C deficiency may become clinically visible, with manifestations such as impaired wound healing, perifollicular hemorrhage, bruising, poor collagen integrity, and increased tissue fragility.

Topical vitamin C has an established dermatologic research base related to photoprotection, pigmentation, and skin aging, whereas systemic vitamin C status remains relevant to dermal collagen metabolism and wound healing, particularly in patients with poor intake, smoking exposure, chronic wounds, pressure injuries, postoperative wounds, cosmetic procedures, or inflammatory skin conditions associated with oxidative stress [42].

Systemic vitamin C is most defensible in dermatologic care when low intake, collagen turnover, wound repair, smoking exposure, or procedural recovery is central to management. It should be paired with photoprotection, adequate protein intake, zinc, vitamin D, glycemic control, and smoking cessation.

### 4.8. Vascular, Metabolic, and Environmental Stress

In vascular and metabolic disease, the relevant issue is endothelial vulnerability under oxidative stress rather than a specific cardiovascular treatment effect. Vitamin C supplementation may improve endothelial measures in selected higher-risk groups, whereas effects are less consistent in healthy individuals with adequate intake [43].

The most defensible interpretation is potential endothelial redox support in smokers, patients with diabetes or metabolic syndrome, and patients with poor diet quality or pollutant exposure. Current evidence is most consistent with endothelial redox support in individuals with increased oxidative burden, but it does not justify a disease-modifying cardiovascular or metabolic claim [44]. A recent umbrella review of randomized-trial meta-analyses on blood pressure also supports cautious interpretation, because statistically significant changes in cardiometabolic surrogate outcomes may be small, heterogeneous, or insufficient to establish a disease-modifying cardiovascular effect [45].

Modern environmental and metabolic contexts may increase oxidative stress and vulnerability to suboptimal micronutrient status. Air pollution, heavy metals, tobacco exposure, chronic hyperglycemia, obesity-related inflammation, and processed dietary patterns may all affect redox balance. Vitamin C has been studied in relation to toxicant exposure and oxidative stress; however, exposure reduction, treatment of toxicity when present, and correction of nutritional inadequacy remain the primary strategies.

In metabolic disease, vitamin C status may be influenced by inflammation, oxidative stress, renal handling, dietary quality, and competition with glucose-related transport or cellular uptake pathways. Patients with diabetes or metabolic syndrome may therefore represent a group in whom vitamin C adequacy deserves attention, although supplementation should not be framed as a glucose-lowering or disease-modifying metabolic therapy [46]. Recent meta-analytic evidence in type 2 diabetes suggests that vitamin C-containing antioxidant supplementation may influence glycemic, lipid, or blood-pressure-related outcomes; however, interpretation remains limited by co-supplementation with vitamin E in some studies, baseline metabolic status, dose, duration, and between-study heterogeneity [47].

### 4.9. Summary of Clinical Domains

Across orthopedic recovery, pain, wound healing, cancer-supportive care, fatigue, neuropsychiatric symptoms, skin biology, and vascular or environmental stress, vitamin C is most relevant when poor intake or vulnerability to low-vitamin C status overlaps with collagen biology, redox balance, immune function, endothelial homeostasis, or tissue repair. The main clinical domains, biological rationale, recommended framing, evidence-sensitive interpretation, and key cautions are summarized in Table 1. Representative clinical evidence across major domains, including study type, population, route, dose pattern, main outcomes, and evidence-sensitive interpretation, is summarized in Supplementary Table S1.

## 5. Patient Selection: When Vitamin C’s Relevance May Increase

Vitamin C consideration begins with patient selection rather than dose selection. Although vitamin C is essential for all humans, supplementation beyond routine dietary intake is most defensible when inadequate intake, impaired absorption, increased oxidative or inflammatory burden, or tissue-repair demand makes suboptimal status more likely. In this review, increased vitamin C demand is not defined as a formal diagnostic category or as an automatic indication for intensified supplementation. Rather, it refers to clinical situations in which reduced intake, impaired absorption, increased metabolic turnover, transient depletion during acute stress, or increased vulnerability to suboptimal status may coexist. Clinically, this vulnerability can be inferred from dietary history, clinical context, risk factors, baseline plasma vitamin C status when available, renal function, inflammatory burden, and the intended clinical endpoint. Clinically, correction of established deficiency, prevention of insufficiency in high-risk individuals, and supplementation intended to address increased physiological demand should be distinguished. Increased physiological demand may reflect increased metabolic turnover or transient depletion during acute stress rather than a formally defined state requiring automatic high-dose supplementation. Therefore, such cases should prompt nutritional assessment, baseline vitamin C evaluation when available, and individualized risk–benefit review.

Population-level recommendations are designed to prevent deficiency in generally healthy individuals. Clinical practice often involves patients with trauma, surgery, chronic inflammation, malignancy, metabolic disease, fatigue, neuropsychiatric symptoms, smoking exposure, poor diet quality, polypharmacy, or environmental stress. These patients may not have overt scurvy, but they may be more vulnerable to low or suboptimal vitamin C status.

Supplementation decisions therefore require individualization according to nutritional risk, clinical stress load, baseline status when available, gastrointestinal tolerance, renal risk, route of administration, and therapeutic context.

### 5.1. Poor Intake, Restricted Diets, and Nutritional Vulnerability

The most straightforward reason to assess vitamin C status or intake is inadequate dietary intake. Patients with low fruit and vegetable consumption, restrictive diets, food insecurity, eating disorders, alcohol use disorder, frailty, institutionalization, poor appetite, nausea, dysphagia, gastrointestinal illness, or cancer-related anorexia may have insufficient vitamin C intake [48,49,50].

In these patients, vitamin C supplementation is best understood first as nutritional repletion. Low-to-moderate dosing may be sufficient when the goal is to correct inadequate intake. High-dose oral supplementation is not automatically required. Clinicians also need to assess broader nutritional status, including protein intake, total caloric intake, vitamin D, B vitamins, magnesium, zinc, iron status, and sarcopenia risk.

Older adults are particularly vulnerable because appetite, dentition, mobility, socioeconomic factors, medication burden, and chronic disease can reduce dietary quality. In frail or institutionalized patients, subclinical vitamin C insufficiency may contribute to fatigue, poor wound healing, bruising, low mood, and reduced physiologic resilience. In these settings, vitamin C belongs within comprehensive nutritional support.

### 5.2. Smoking, Secondhand Smoke, and Environmental Oxidative Burden

Smoking is one of the clearest examples of exposure-related vulnerability to low-vitamin C status because tobacco smoke increases oxidative burden and is associated with lower vitamin C status [1,51]. Smokers may therefore require higher intake than nonsmokers to achieve comparable vitamin C status, although this should be interpreted as status-oriented nutritional support rather than a disease-directed treatment claim. Dietary reference guidance commonly recommends an additional intake of approximately 35 mg/day above nonsmoker requirements for smokers, reflecting increased oxidative burden rather than a disease-directed treatment effect [14]. Secondhand smoke exposure may also contribute to oxidative burden, although the magnitude is generally lower than active smoking.

Environmental pollutants and heavy metals may similarly increase oxidative stress. Air pollution, fine particulate matter, cadmium, lead, and other toxicant exposures can increase reactive oxygen species generation and may increase vulnerability to antioxidant micronutrient inadequacy. Vitamin C has been studied as part of antioxidant defense in toxicant exposure; however, supplementation should be clearly separated from exposure reduction, chelation therapy when indicated, and treatment of toxic exposures.

For patients with smoking exposure, occupational pollutant exposure, environmental toxicant burden, or high oxidative stress, vitamin C status or intake deserves review, particularly when dietary intake is poor. Smoking cessation, exposure reduction, and appropriate toxicity treatment remain the primary interventions.

### 5.3. Infection, Trauma, Surgery, and Acute Inflammatory Stress

Acute illness, trauma, and surgery may accelerate vitamin C turnover through immune activation, tissue injury, wound healing, endothelial stress, and systemic inflammation; postoperative plasma vitamin C concentrations have been reported to decline after surgery [52,53]. In critically ill or postoperative patients, vitamin C levels may decline rapidly, reflecting increased consumption, redistribution, reduced intake, and metabolic stress.

In surgical and orthopedic patients, vitamin C relevance may increase in the context of wound healing, collagen synthesis, bleeding, inflammation, immobilization, pain, and rehabilitation-related tissue remodeling. Patients undergoing fracture fixation, tendon or ligament repair, joint surgery, spine procedures, soft-tissue surgery, or wound management may therefore be reasonable candidates for assessment and maintenance of adequate vitamin C status.

Perioperative and post-injury care should position vitamin C within nutritional optimization, alongside surgical technique, infection control, fixation stability, wound care, rehabilitation, glycemic control, and adequate protein intake. In trauma, surgery, and tissue-repair settings, maintaining adequate vitamin C status should be distinguished from claiming that additional supplementation improves clinical outcomes. Evidence is most defensible for correcting or preventing inadequate status in nutritionally vulnerable patients, whereas clinically meaningful improvements in healing, pain, radiographic union, or functional recovery in vitamin C-replete patients remain insufficiently established [9,13].

Oral vitamin C supplementation above conventional maintenance ranges may be considered in selected patients with low intake and substantial inflammatory or tissue-repair burden, but this approach is based mainly on pharmacokinetic rationale, nutritional-risk assessment, mechanistic plausibility, and limited condition-specific clinical evidence rather than a universal randomized trial-defined dose target [7,9,13,51]. When used, renal risk should be low, and dosing should be individualized, time-limited, divided, and guided by gastrointestinal tolerability and safety monitoring. In uncomplicated patients with adequate intake, conventional supplementation may be sufficient. When doses above conventional supplemental ranges are considered, divided oral dosing is generally more appropriate than large bolus dosing.

### 5.4. Orthopedic Recovery, Fracture Healing, and Regenerative Procedures

Orthopedic patients often have overlapping reasons to assess vitamin C status, including tissue injury, collagen remodeling, postoperative recovery, smoking, diabetes, and delayed wound healing [13]. Vitamin C adequacy may be particularly relevant to the nutritional context of fracture healing, tendon injury, ligament injury, enthesopathy, fasciopathy, wound healing, pressure injury, and postoperative soft-tissue recovery.

In fracture patients, vitamin C is relevant to collagen formation, callus biology, oxidative-stress regulation, and selected CRPS-I risk-reduction contexts. The evidence for CRPS risk reduction is more clinically developed than the evidence for accelerated radiographic union, and these outcomes should be interpreted separately.

In tissue-repair-oriented interventions, including prolotherapy, extracorporeal shockwave therapy, platelet-rich plasma, and other regenerative procedures, vitamin C should be viewed as part of the nutritional milieu that may support collagen remodeling and matrix repair rather than as the regenerative intervention itself. The primary therapeutic effects of these procedures arise from local mechanical, biologic, or injection-mediated mechanisms, whereas vitamin C mainly supports cofactor-dependent repair biology when nutritional status is suboptimal or repair demand is high. Prolotherapy reviews support the concept of injection-mediated stimulation of connective tissue repair [54,55]. A multicomponent nutraceutical adjunct containing vitamin C has been studied alongside extracorporeal shockwave therapy in chronic tendinopathy [56]. Because the intervention included multiple active ingredients and was combined with ESWT, the observed outcomes cannot be attributed specifically to vitamin C. These data should therefore be interpreted as adjunctive and hypothesis-generating rather than as evidence of vitamin C-specific regenerative efficacy. Case-level orthopedic reports have also described tissue-repair-oriented interventions combined with adjunctive nutritional and metabolic support in calcific tendinopathy, but such observations remain hypothesis-generating and cannot establish vitamin C-specific efficacy [57].

Patients requiring closer nutritional attention include smokers, older adults, patients with diabetes or poor diet quality, those with delayed wound healing or recurrent tendon or ligament problems, and those undergoing repeated regenerative or postoperative recovery protocols. Vitamin C belongs in the same discussion as protein intake, vitamin D, magnesium, zinc, sleep, glycemic control, and appropriate mechanical loading.

### 5.5. Cancer, Cancer Treatment, and Cachexia-Related Vulnerability

In oncology, patient selection requires caution and coordination because oral nutritional supplementation and intravenous pharmacologic ascorbate differ in purpose, pharmacokinetics, monitoring requirements, and evidentiary status [32]. Oral vitamin C is most appropriately considered for nutritional repletion in patients with poor intake, fatigue, treatment-related symptoms, or increased inflammatory burden. Intravenous vitamin C is a separate pharmacologic intervention requiring medical oversight, safety screening, and coordination with the oncology team.

During active chemotherapy or radiation therapy, redox-active interventions require caution because interactions may vary by treatment mechanism, regimen, and timing. In this context, vitamin C use is most appropriately framed around nutritional insufficiency, fatigue, postoperative recovery, quality-of-life support, or other adjunctive care goals, with oncology coordination.

Before high-dose oral or intravenous vitamin C is considered in cancer patients, clinicians should consider renal function, hydration, glucose-6-phosphate dehydrogenase (G6PD) status for intravenous therapy, iron metabolism, tumor context, treatment phase, concurrent medications, and the patient’s goals of care.

### 5.6. Fatigue, Neuropsychiatric Symptoms, and Metabolic Vulnerability

Vitamin C status may become clinically informative in nonspecific fatigue, frailty, mood symptoms, cognitive complaints, or reduced activity tolerance when these symptoms coexist with poor intake, smoking, chronic inflammation, cancer-related burden, chronic pain, alcohol use, restrictive diets, or systemic illness [22,34,36,48]. Vitamin C is unlikely to explain these symptoms by itself; rather, vitamin C status belongs within a broader evaluation that includes anemia, thyroid dysfunction, sleep disturbance, depression, medication effects, protein adequacy, sarcopenia, vitamin D, B vitamins, magnesium, glycemic control, and inflammatory or malignant disease. Magnesium status may also be relevant in patients with overlapping neurovascular vulnerability, neuropathic pain, poor sleep, or metabolic disease, and should be considered as part of a broader nutritional assessment rather than as a vitamin C-specific mechanism [58].

Patients with diabetes, obesity, metabolic syndrome, and insulin resistance may also have increased relevance of antioxidant and micronutrient adequacy because oxidative stress, endothelial dysfunction, impaired wound healing, and chronic inflammation often coexist [46]. Related contexts such as metabolic dysfunction-associated steatotic liver disease, dialysis-dependent chronic kidney disease, severe viral infections, and healthy aging may warrant dedicated evaluation in future studies. However, supplementation is better positioned as nutritional support rather than as a primary glucose-lowering or disease-modifying metabolic therapy.

### 5.7. Patients Requiring Caution with High-Dose Use

Patient selection must also identify patients in whom high-dose oral or intravenous vitamin C requires caution, dose limitation, or specialist input. Patients with chronic kidney disease, prior calcium oxalate stones, known hyperoxaluria, oxalate nephropathy history, or severe dehydration risk require caution because high-dose vitamin C may increase oxalate-related renal risk in susceptible patients [59,60]. Patients with iron overload, hereditary hemochromatosis, or repeated transfusion exposure should be distinguished from iron-replete healthy individuals when prolonged high-dose supplementation is considered [25].

For intravenous vitamin C, additional caution is required in patients with G6PD deficiency because high-dose intravenous ascorbate may precipitate hemolysis in susceptible individuals; this caution should not be generalized to conventional oral supplementation [61,62]. Renal function and hydration status should also be reviewed before intravenous therapy.

In patients at higher risk for nephrolithiasis, oral vitamin C is not necessarily contraindicated at nutritional or conventional supplemental doses, but prolonged high-dose use requires individualized risk assessment. Hydration, urinary risk profile, magnesium and vitamin B6 status, dietary oxalate, sodium intake, calcium intake, and renal function require consideration.

### 5.8. Practical Patient-Selection Framework

Patient selection can be organized around four evidence-sensitive questions. First, is there inadequate intake or nutritional vulnerability, such as poor diet quality, restricted intake, frailty, alcohol use, cancer-related anorexia, gastrointestinal symptoms, or institutionalization? Second, is vulnerability to low-vitamin C status increased by trauma, surgery, fracture, wound healing, chronic inflammation, infection, cancer, smoking, environmental exposure, metabolic disease, or oxidative burden? Third, is the clinical goal appropriate, such as correcting deficiency or insufficiency, supporting collagen biology, improving nutritional adequacy, supporting postoperative or post-injury recovery, reducing CRPS risk in selected contexts, or improving fatigue-related care in nutritionally vulnerable patients? Fourth, do safety factors modify dosing, including gastrointestinal sensitivity, kidney stone history, renal impairment, dehydration, iron overload, G6PD deficiency for intravenous therapy, active chemotherapy or radiation therapy, or relevant medication interactions?

Using this approach, vitamin C use can be categorized as nutritional repletion, maintenance supplementation, selected clinician-directed oral supplementation above conventional ranges with monitoring, or intravenous pharmacologic therapy.

A patient-selection summary for vitamin C supplementation or dose escalation in selected contexts is provided in Table 2.

## 6. Practical Clinical Considerations: Route, Formulation, and Dose

Clinical considerations depend on route, formulation, dose unit, dosing interval, gastrointestinal tolerance, and safety context. These factors help distinguish nutritional supplementation, selected clinician-monitored oral dose escalation, and pharmacologic intravenous therapy. The practical considerations in this section are derived from a combination of pharmacokinetic studies, dietary reference guidance, formulation-specific evidence, safety reports, historical clinical observations, mechanistic rationale, and limited clinical outcome data; they should not be interpreted as guideline-level dosing recommendations.

Four categories need distinction: nutritional repletion, maintenance supplementation, selected clinician-directed oral supplementation above conventional ranges, and intravenous pharmacologic therapy. These categories differ in purpose, pharmacokinetics, monitoring requirements, and safety considerations.

### 6.1. Oral Versus Intravenous Vitamin C

Oral and intravenous vitamin C should be treated as different interventions because oral dosing is constrained by regulated absorption and renal handling, whereas intravenous dosing can achieve pharmacologic plasma concentrations [7,8]. Oral dosing is suited to nutritional repletion, maintenance supplementation, redox support, collagen turnover, immune support, and, in selected monitored contexts, divided dosing above conventional supplemental ranges guided by gastrointestinal tolerability. IV dosing bypasses intestinal absorption limits and produces pharmacologic plasma concentrations that ordinary oral dosing cannot achieve.

Oral vitamin C absorption is dose-dependent and saturable, largely reflecting regulated intestinal transport and renal elimination [51]. Fractional absorption is high at lower doses, but it decreases at gram-level doses as plasma and tissue handling approach saturation and urinary excretion rises. This does not establish clinical superiority of high-dose oral use; rather, it makes dose division and tolerability central when doses above conventional supplemental ranges are considered. Large single boluses leave more unabsorbed ascorbate in the intestinal lumen and can increase osmotic gastrointestinal symptoms.

Intravenous ascorbate is a medical intervention rather than a nutritional supplement and should be limited to defined contexts, such as cancer-supportive care, critical illness research, or severe deficiency requiring rapid correction, with appropriate medical supervision. Renal function, hydration status, G6PD deficiency, iron-overload risk, and concurrent oncologic therapy require review before high-dose IV use.

This distinction prevents dietary supplementation from being extrapolated to pharmacologic infusion therapy.

### 6.2. Oral Preparations: Tablets, Capsules, Powders, and Liquids

Oral vitamin C products vary in practical usability. Tablets and capsules are convenient, portable, and dose-standardized, making them useful for routine maintenance supplementation. However, they require disintegration and dissolution before absorption. Large tablets may be difficult for some patients to swallow and may cause gastric discomfort, particularly when repeated gram-level dosing is considered.

Powder formulations often provide greater flexibility when oral dose adjustment is needed because they allow smaller divided doses and mixing with sufficient fluid. This is particularly useful when dosing is limited by gastrointestinal tolerability. If loose stool, bloating, abdominal cramping, nausea, or reflux occurs, the patient can reduce the dose or increase the dosing interval without changing the overall nutritional support plan.

Liquid preparations or fully dissolved powders may be useful for patients with dysphagia, poor adherence to tablets, postoperative swallowing difficulty, or the need for more rapid ingestion. However, vitamin C is sensitive to light, oxygen, heat, pH, and metal ions. Therefore, liquid products and pre-dissolved preparations require attention to storage, stability, and timing of ingestion.

Form selection should follow the intended use: tablets or capsules for maintenance, powders or dissolved forms when flexible divided dosing is needed, and liquid or dissolved preparations for dysphagia or poor tolerance of large tablets.

### 6.3. Fine, Ultrafine, and Crystalline Oral Forms

Commercial ascorbic acid products may differ in particle size and physical form, including fine powder, ultrafine powder, and coarser crystalline preparations. The claim that crystalline ascorbic acid is “not absorbed” is inaccurate because L-ascorbic acid is water soluble and can be absorbed after adequate dissolution [14,51]. Coarser crystalline preparations may dissolve more slowly and may be less convenient for repeated divided dosing, particularly when taken as large boluses or without sufficient fluid. Smaller-particle preparations may improve dispersion, mixing, and practical usability, but direct pharmacokinetic or clinical outcome data comparing fine, ultrafine, and coarse crystalline ascorbic acid remain limited. Therefore, particle-size claims should be limited to dissolution, tolerability, and adherence rather than bioavailability superiority or disease-specific efficacy [7,51,63].

### 6.4. Buffered, Mineral, Sustained-Release, and Liposomal Formulations

Ascorbic acid is acidic and may cause gastric discomfort in some patients, especially when taken as repeated or large single doses. Buffered or mineral ascorbate preparations, including sodium ascorbate, calcium ascorbate, magnesium ascorbate, or mixed mineral ascorbates, may improve gastric tolerability in acid-sensitive patients. However, the mineral component must be considered clinically.

Sodium ascorbate may be less acidic but adds sodium load, which may be relevant in patients with hypertension, heart failure, renal disease, or sodium restriction. Calcium ascorbate adds calcium, which may be undesirable in patients with hypercalcemia, calcium stone risk, or specific metabolic concerns. Magnesium ascorbate may be useful in patients with concurrent magnesium insufficiency, but renal function matters when magnesium intake is increased. Buffered formulations may improve tolerance, but comorbidities and mineral load should guide selection.

Sustained-release products may alter plasma exposure compared with plain ascorbic acid, but available pharmacokinetic studies are formulation-specific and should not be generalized to all products [64,65]. They may be useful for low-to-moderate maintenance supplementation, but they are less flexible when rapid gastrointestinal tolerance-limited dose adjustment is needed. When dose adjustment is limited by gastrointestinal symptoms, immediate-release powder or dissolved forms may be preferable because they allow more flexible adjustment.

Liposomal vitamin C refers to ascorbate incorporated into phospholipid vesicles or lipid-based carriers [66,67,68]. Some products may alter pharmacokinetic exposure or gastrointestinal tolerability, but studies vary in composition, encapsulation efficiency, particle size, phospholipid source, stability, and sampling design, and clinical outcome data remain limited. Findings from one product therefore should not be generalized to all lipid-based preparations. The term “liposomal” also does not define the source or quality documentation of the ascorbic acid; manufacturing source is best discussed in terms of reproducibility, impurity control, regulatory standards, and certificate-of-analysis quality rather than assumed clinical superiority [63].

Across oral formulations, improved dissolution, gastric tolerability, or pharmacokinetic exposure should not be assumed to imply clinically meaningful superiority unless supported by direct comparative clinical outcome data.

### 6.5. Dose Considerations: Total Daily Dose Versus Single-Dose Unit

A clinically important distinction is total daily dose versus single-dose unit. Much of the concern around oral vitamin C dose escalation arises when total daily intake is discussed without clarifying how the dose is divided, why dose escalation is being considered, and how safety is monitored. A patient receiving an oral dose above conventional supplemental ranges should not receive the entire total daily dose as a single bolus. Large single boluses are generally less rational than divided dosing because intestinal vitamin C absorption is saturable and gastrointestinal intolerance is dose-dependent.

Oral ascorbate uptake is mediated in part by sodium-dependent vitamin C transporters, particularly SVCT1 in the intestine. As single-dose size increases, fractional absorption decreases and urinary excretion increases, making divided dosing more pharmacokinetically rational than large single boluses, and more unabsorbed ascorbate may remain in the intestinal lumen [7,51]. This can increase osmotic effects and lead to loose stool, bloating, cramping, nausea, or diarrhea. Therefore, when oral dose escalation is considered, the goal is not to force the largest possible single dose, but to maintain tolerable divided exposure while minimizing gastrointestinal loss and intolerance.

In selected monitored contexts, approximately 1–2 g per dose may be a practical divided-dose unit, although this should not be interpreted as an evidence-defined therapeutic dose. Patients with gastrointestinal sensitivity may start with lower single doses, such as 500 mg to 1 g per dose. Patients who tolerate supplementation well may use divided doses adjusted according to clinical context, safety factors, and gastrointestinal tolerability. Current evidence does not establish a universal total daily target above conventional supplemental ranges; therefore, dose escalation, if used, should remain individualized, time-limited, divided, and clinician-monitored rather than presented as a fixed therapeutic range.

Large single doses are less suitable for routine oral use because they are more likely to reduce fractional absorption and increase gastrointestinal symptoms. Divided dosing better matches transporter-limited absorption and may improve tolerability, but it does not by itself establish superior clinical efficacy.

### 6.6. Gastrointestinal Tolerance-Limited Dose Adjustment and the Historical Bowel-Tolerance Concept

The bowel-tolerance concept is a historical clinical model most closely associated with Cathcart’s work on high-dose oral vitamin C [69]. In this model, oral vitamin C is increased in divided doses until loose stool or diarrhea occurs, after which the dose is reduced to a tolerated level. Cathcart observed that bowel tolerance appeared to increase during infection, trauma, surgery, inflammation, and malignancy, but these observations were not derived from contemporary controlled trials, standardized outcome measures, pharmacokinetic validation, or prospective safety assessment.

In contemporary practice, gastrointestinal tolerance should be regarded as an adverse-effect boundary, not as a validated biomarker of systemic requirement or therapeutic adequacy. Gastrointestinal symptoms serve as a safety signal, and any dose adjustment requires patient education, hydration, renal-risk assessment, and periodic reassessment. Its main practical value is that it discourages rigid dose escalation and reinforces dose reduction when gastrointestinal intolerance occurs.

When oral dose escalation is used, escalation should stop when loose stool, cramping, bloating, nausea, reflux, or diarrhea occurs. The dose should then be reduced to a previously tolerated dose or supplementation should be discontinued if symptoms persist. This approach should be interpreted as gastrointestinal tolerance-limited safety adjustment rather than requirement-based titration.

### 6.7. Relationship with the Tolerable Upper Intake Level

The adult tolerable upper intake level for vitamin C is commonly cited as 2 g/day [1]. This value is important for public health guidance and unsupervised routine supplementation, and it should remain the primary benchmark for general use. The upper intake level is primarily based on the risk of gastrointestinal disturbance, especially osmotic diarrhea, and should not be interpreted as proof that higher intakes are clinically beneficial in healthy individuals.

The upper intake level remains a conservative benchmark for unsupervised supplementation. Clinician-directed oral use above the upper intake level belongs to a different category and requires a defined clinical rationale, patient selection, divided dosing, gastrointestinal tolerability monitoring, hydration, renal-risk assessment, and periodic reassessment.

For the general public, routine unsupervised intake above 2 g/day is unnecessary and may cause gastrointestinal symptoms. In selected monitored clinical contexts, intakes above the upper intake level may be considered when nutritional vulnerability or increased clinical stress is present and safety factors are addressed, but no universal high-dose target has been established.

### 6.8. Transition to Safety and Monitoring

Route, formulation, dose unit, dosing interval, and duration influence gastrointestinal tolerance, renal risk, hydration requirements, iron handling, and oncology coordination. Route, formulation, and dosing considerations for clinical vitamin C use are summarized in Table 3; safety and monitoring are addressed in the following section.

## 7. Safety and Risk Management

Safety review should be proportional to dose, duration, route, baseline vulnerability, and clinical context. Conventional oral supplementation is generally well tolerated, whereas prolonged oral supplementation above conventional ranges or intravenous pharmacologic ascorbate requires closer attention to gastrointestinal tolerance, renal function, stone history, iron-overload risk, G6PD status for intravenous therapy, active oncologic treatment, and selected laboratory interactions [14,70].

### 7.1. Gastrointestinal Intolerance

The most common adverse effects of oral vitamin C dose escalation are gastrointestinal, including loose stool, diarrhea, abdominal cramping, bloating, nausea, reflux, and gastric irritation [14]. These symptoms are usually dose related, reversible, and more likely after large single boluses than after smaller divided doses. When gastrointestinal symptoms occur, the dose should be reduced, the dosing interval should be lengthened, or supplementation should be discontinued if symptoms persist. Persistent diarrhea, dehydration, abdominal pain, or intolerance despite dose reduction should prompt discontinuation, hydration review, and clinical reassessment.

### 7.2. Nephrolithiasis and Oxalate Concerns

Vitamin C can be metabolized to oxalate, and oral supplementation above conventional ranges may increase urinary oxalate in some individuals [71]. Whether this biochemical change translates into clinically meaningful nephrolithiasis risk depends on patient susceptibility, sex, hydration, urinary chemistry, renal function, diet, dose, formulation, and duration [59]. Patients with prior calcium oxalate stones, known hyperoxaluria, chronic kidney disease, dehydration risk, malabsorption, bariatric surgery history, or recurrent nephrolithiasis require individualized risk assessment and, when appropriate, renal function testing, 24-h urine stone risk evaluation, or nephrology/urology consultation.

Cases of oxalate nephropathy have been reported in medically complex patients exposed to very-high-dose vitamin C, particularly intravenously or in the setting of renal impairment, critical illness, dehydration, or other concurrent risk factors [60,70]. These cases should not be generalized to ordinary oral supplementation, but they support renal-risk review before prolonged oral supplementation above conventional ranges or intravenous therapy.

### 7.3. Iron Overload and Hemochromatosis

Vitamin C can enhance non-heme iron absorption; therefore, prolonged oral supplementation above conventional ranges or intravenous pharmacologic ascorbate requires caution in patients with hereditary hemochromatosis, repeated transfusion exposure, selected hematologic disorders, or markedly elevated ferritin with high transferrin saturation [25]. Routine iron testing is not necessary before ordinary supplementation in healthy individuals, but ferritin, transferrin saturation, liver disease status, transfusion history, and hematologic diagnosis should guide decisions when iron-overload risk is present or prolonged high-dose use is being considered.

### 7.4. Glucose-6-Phosphate Dehydrogenase Deficiency and Intravenous Vitamin C

G6PD deficiency is primarily relevant to high-dose intravenous vitamin C because hemolysis has been reported in susceptible patients exposed to pharmacologic ascorbate concentrations; this concern should not be extrapolated to conventional oral supplementation [62]. Conventional oral supplementation should therefore not be treated as equivalent to high-dose intravenous ascorbate. For intravenous therapy, G6PD status, renal function, hydration status, and the clinical indication should be reviewed before treatment.

### 7.5. Oncology-Specific Safety Considerations

In cancer care, oral supplementation above conventional ranges or intravenous pharmacologic ascorbate should remain aligned with nutritional or symptom-supportive goals and should not be presented as a substitute for standard oncologic therapy [10,32]. During active chemotherapy, radiation therapy, immunotherapy, or targeted therapy, vitamin C use beyond routine nutritional repletion should be coordinated with the treating oncologist because regimen-specific redox interactions, renal risk, treatment timing, and patient goals of care may affect safety decisions.

High-dose intravenous vitamin C and, less commonly, high oral exposures may interfere with selected point-of-care glucose measurements depending on device methodology, especially in critically ill, diabetic, or intravenously treated patients [72,73]. Clinicians should consider laboratory confirmation when point-of-care glucose readings are inconsistent with the clinical picture.

Recent systematic review evidence on high-dose intravenous vitamin C further supports separating pharmacologic IV exposure from oral supplementation when interpreting potential benefit and route-specific safety concerns, including renal risk, G6PD-related hemolysis, and laboratory interference [74].

### 7.6. Special Populations and Monitoring

Pregnancy, lactation, children, adolescents, frail older adults, and patients with chronic kidney disease require individualized dosing because reference intakes, upper intake levels, renal reserve, medication burden, and swallowing ability differ by life stage and clinical condition. Any use above conventional supplemental ranges should have a defined clinical purpose, a dose-adjustment plan, a safety review, and periodic reassessment. Dose reduction to maintenance supplementation or dietary adequacy is appropriate once the period of nutritional vulnerability or increased clinical stress has passed [14].

## 8. Practical Clinical Decision Framework

The practical decision framework begins with purpose rather than dose. Use can be classified as nutritional repletion, maintenance supplementation, selected clinician-directed oral supplementation above conventional ranges, or intravenous pharmacologic therapy, and these categories should not be treated as interchangeable [1,8].

Once the purpose is defined, clinicians should assess whether nutritional vulnerability and increased clinical stress are present rather than assuming there is a need for dose escalation. Relevant factors include poor fruit and vegetable intake, smoking, infection, trauma, surgery, fracture, chronic wounds, postoperative recovery, cancer, active cancer treatment, metabolic disease, chronic inflammation, frailty, fatigue, and neuropsychiatric vulnerability. Easy bruising, gum bleeding, delayed wound healing, recurrent infection, low mood, fatigue, and poor dietary quality may support further nutritional assessment or baseline vitamin C evaluation when available.

Safety screening should cover gastrointestinal sensitivity, stone history, renal impairment, dehydration risk, iron overload, active oncologic therapy, and G6PD deficiency when intravenous therapy is being considered. G6PD-related hemolysis risk should be framed primarily in relation to high-dose intravenous ascorbate rather than conventional oral supplementation.

Route and formulation should then be matched to the clinical goal. Oral dosing is appropriate for nutritional repletion, maintenance supplementation, collagen-related nutritional support, and selected divided dosing when supplementation above conventional ranges is considered. Intravenous therapy should be reserved for situations in which pharmacologic plasma concentrations are intended or oral intake is not feasible. Tablets or capsules may be sufficient for routine supplementation, whereas powders or dissolved forms may be more useful when flexible divided dosing is needed. Buffered or mineral ascorbates may be used in acid-sensitive patients when the mineral load is clinically appropriate.

When oral supplementation above conventional ranges is considered, clinicians should distinguish the single-dose unit from total daily intake because oral absorption and renal handling are dose-dependent [7,51]. Patients with gastrointestinal sensitivity may start with lower single doses, such as 500 mg to 1 g per dose. In selected monitored contexts, approximately 1–2 g per dose may be a practical divided-dose unit, but this should not be interpreted as an evidence-defined therapeutic dose. Current evidence does not establish a universal high-dose oral target for stress-responsive populations. If oral dose escalation above conventional supplemental ranges is considered, it should be individualized, time-limited, divided, and clinician-monitored. Escalation should stop at loose stool, bloating, cramping, nausea, reflux, or diarrhea, followed by dose reduction or discontinuation if symptoms persist.

**Figure 2:**
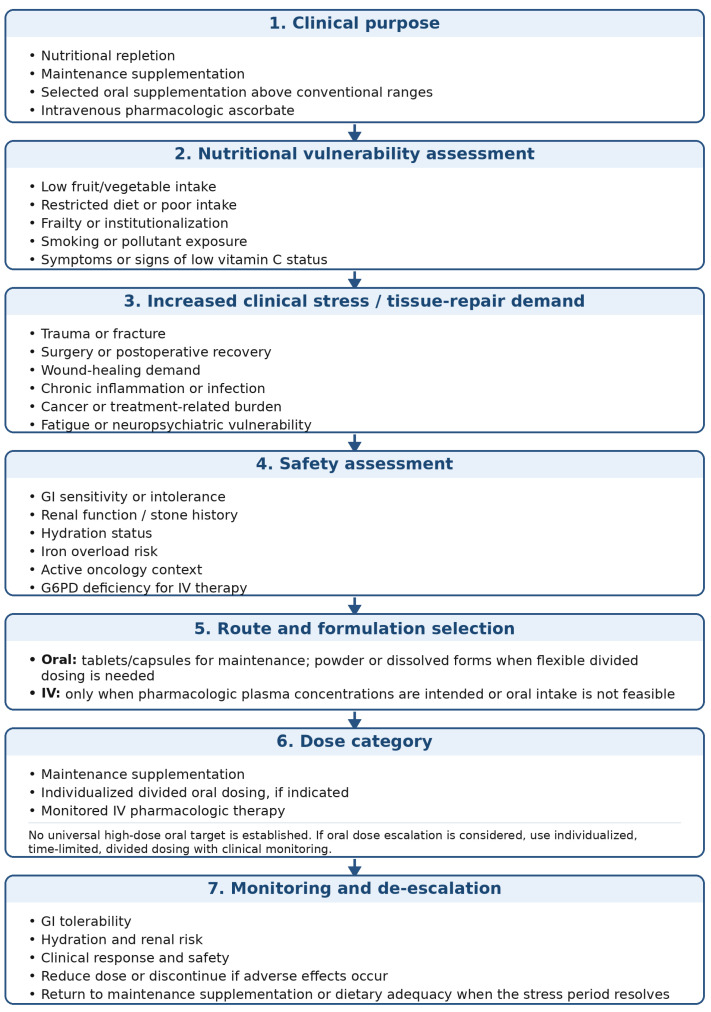
Practical clinical decision framework for vitamin C use. The framework begins with clinical purpose rather than dose and separates nutritional repletion, maintenance supplementation, selected clinician-directed oral supplementation above conventional ranges, and intravenous pharmacologic ascorbate. Oral dose escalation should not be interpreted as a universal recommendation or evidence-defined therapeutic target and should be individualized according to nutritional vulnerability, clinical stress, route, formulation, gastrointestinal tolerability, renal risk, iron status, active oncologic context, and monitoring feasibility. Abbreviations: GI, gastrointestinal; G6PD, glucose-6-phosphate dehydrogenase; IV, intravenous.

Monitoring and de-escalation should follow the safety principles summarized in Section 7, with return to maintenance supplementation or dietary adequacy once the period of nutritional vulnerability or increased clinical stress has resolved. When clinically available, monitoring may also incorporate baseline or follow-up vitamin C status, renal function, inflammatory markers such as C-reactive protein, urinary stone-risk assessment in susceptible patients, and validated domain-specific clinical outcomes. These measures should complement, not replace, clinical judgment. To improve clinical transparency, this decision framework is summarized in Figure 2. The framework is intended to support structured clinical reasoning rather than to function as a disease-specific guideline. It emphasizes clinical indication, nutritional vulnerability, safety assessment, route and formulation selection, dose category, gastrointestinal tolerability, monitoring, and de-escalation.

## 9. Limitations

As a narrative synthesis, this review did not include a formal systematic search protocol, risk-of-bias assessment, evidence grading, or quantitative analysis. The included literature spans mechanistic studies, pharmacokinetic investigations, clinical trials, observational studies, systematic reviews, dietary reference documents, and historically influential clinical models. Accordingly, the conclusions should be interpreted as a clinically oriented narrative synthesis rather than guideline-level evidence or formal clinical recommendations [11].

The available clinical literature is highly heterogeneous. Studies differ substantially in baseline vitamin C status, population characteristics, route of administration, formulation, dose, dosing interval, treatment duration, clinical endpoints, comparator selection, and safety monitoring. This heterogeneity is especially relevant in studies of fatigue, cancer-supportive care, chronic pain, wound healing, musculoskeletal recovery, and neuropsychiatric symptoms. Many studies do not measure baseline vitamin C status, making it difficult to distinguish the correction of deficiency or insufficiency from pharmacologic, contextual, or nonspecific treatment effects in nutritionally sufficient individuals [13,51].

A major limitation of the existing literature is that some studies and discussions group oral and intravenous ascorbate as if they were the same intervention [8]. Oral dosing is constrained by saturable intestinal absorption and is best suited to nutritional repletion, maintenance supplementation, redox support, collagen turnover, and selected divided dosing when supplementation above conventional ranges is considered. IV dosing bypasses intestinal regulation and produces pharmacologic plasma concentrations. This distinction limits direct extrapolation across studies unless the intended exposure is clearly defined as nutritional repletion, supportive care, or pharmacologic ascorbate.

Formulation-specific clinical evidence also remains limited [68]. Tablets, powders, liquids, buffered preparations, mineral ascorbates, sustained-release products, liposomal products, and fine, ultrafine, or crystalline forms may differ in dissolution, tolerability, adherence, plasma exposure, and dosing flexibility. However, direct comparative pharmacokinetic and clinical outcome studies remain scarce. Future formulation studies should report key product characteristics, including particle size, dissolution behavior, encapsulation efficiency when applicable, phospholipid source, stability data, source documentation, and certificate-of-analysis standards. Claims regarding liposomal delivery, sustained release, particle grade, raw-material source, or enhanced absorption therefore require transparent pharmacokinetic methods and, when possible, clinically relevant outcome data.

The historical bowel-tolerance model also remains insufficiently validated. Cathcart’s observations remain historically influential in high-dose oral vitamin C practice, but bowel tolerance has not been validated as a standardized biomarker of systemic requirement, therapeutic adequacy, or clinical efficacy [69]. Prospective studies should evaluate divided dosing, larger bolus dosing, gastrointestinal tolerance-limited adjustment, plasma and leukocyte vitamin C concentrations, urinary oxalate, renal safety, and clinical outcomes under controlled conditions.

Long-term safety data for prolonged oral supplementation above conventional ranges remain incomplete, especially in stone-prone patients, chronic kidney disease, metabolic disease, and medically complex cancer care [59,70]. Nephrolithiasis risk is difficult to isolate because stone formation depends on urine volume, oxalate, calcium, citrate, magnesium, sodium, uric acid, pH, diet, renal function, hydration, and genetic or metabolic predisposition. Safety interpretation is limited when studies do not report renal function, stone history, urinary chemistry, dose duration, hydration status, and concomitant risk modifiers such as magnesium, vitamin B6, citrate, sodium intake, and dietary oxalate.

## 10. Future Directions

Future research should move from broad supplementation claims toward predefined patient populations, baseline vitamin C assessment, route-specific exposure definitions, formulation transparency, dose rationale, treatment duration, clinical endpoints, and safety monitoring. Multicenter randomized trials are needed to determine which populations benefit from vitamin C supplementation, whether benefits depend on baseline deficiency or insufficiency, which doses and durations are clinically meaningful, and which outcomes are reproducible across settings. Precision nutrition approaches should evaluate how age, sex, frailty, inflammatory status, metabolic disease, renal function, gut microbiota, genetic factors, medication burden, smoking exposure, and baseline vitamin C concentrations modify response to supplementation. Such studies may help distinguish patients who require nutritional repletion from those unlikely to benefit from supraphysiologic exposure.

Biomarker-guided studies are also needed. Future trials should consider plasma vitamin C concentrations, leukocyte vitamin C concentrations when feasible, inflammatory markers, oxidative-stress biomarkers, renal function, urinary oxalate, metabolomic profiles, microbiome features, and validated clinical outcomes. This would allow efficacy and safety to be interpreted within a biologically defined patient context rather than by dose alone.

Formulation research should directly compare conventional ascorbic acid, buffered or mineral ascorbates, sustained-release products, liposomal preparations, powders, dissolved forms, and intravenous ascorbate using transparent product characterization and pharmacokinetic methods. Clinically relevant outcomes should be included whenever possible, because improved exposure or tolerability does not necessarily imply superior disease-related efficacy.

Additional clinical research is needed in oncology, orthopedic recovery, regenerative medicine, fatigue, and neuropsychiatric symptoms. Cancer-related studies should distinguish nutritional, supportive, pharmacologic, and disease-modifying endpoints. Orthopedic and regenerative studies should evaluate fracture recovery, tendon and ligament injury, postoperative wound healing, selected CRPS-risk contexts, and tissue-repair-oriented interventions while accounting for protein intake, vitamin D, magnesium, zinc, glycemic control, smoking, sleep, mechanical loading, and rehabilitation. Fatigue and neuropsychiatric studies should define patient subgroups according to baseline vitamin C status, diet quality, inflammation, smoking, cancer burden, chronic pain, frailty, and systemic illness. Future research should also evaluate vitamin C within multimodal nutritional and lifestyle strategies, including protein or hydrolyzed collagen intake, vitamin D, magnesium, zinc, polyphenols, gut microbiota modulation, physical exercise, healthy aging, and regenerative or rehabilitation-based care.

Overall, future studies need predefined patient populations, baseline vitamin C assessment, route-specific exposure definitions, formulation characterization, dose rationale, clinical endpoints, and monitoring plans before efficacy or safety can be interpreted.

## 11. Conclusions

Vitamin C is more than a deficiency-prevention nutrient. It participates in collagen biosynthesis, extracellular matrix stability, redox regulation, immune function, endothelial biology, carnitine synthesis, neurotransmitter metabolism, skin integrity, fatigue-related physiology, and tissue repair. These functions may become clinically relevant when poor intake, oxidative or inflammatory burden, malignancy, pharmacologic stress, environmental exposure, or tissue-repair demand increases vulnerability to low- or suboptimal vitamin C status.

In orthopedic and regenerative practice, vitamin C is best framed as a nutritional cofactor for collagen maturation, extracellular matrix remodeling, wound healing, and tissue repair rather than as an independent regenerative intervention. In pain care, the strongest clinical signal relates to selected CRPS-risk contexts after fracture or extremity surgery, although evidence remains mixed and should not be generalized to all pain conditions. In cancer-supportive care, oral nutritional supplementation and intravenous pharmacologic ascorbate require clear separation and coordination with standard oncologic treatment. In fatigue and neuropsychiatric symptoms, vitamin C is most relevant when deficiency, poor diet quality, inflammation, systemic illness, or increased oxidative stress is present.

Clinical use should therefore be guided by baseline status when available, route, formulation, dose unit, dosing interval, gastrointestinal tolerability, renal risk, iron status, hydration, duration, and oncologic setting. Divided oral dosing may improve tolerability when supplementation above conventional ranges is considered, but current evidence does not establish a universal high-dose oral target for stress-responsive populations. Gastrointestinal tolerance should be interpreted as a safety boundary rather than a validated biomarker of systemic requirement or therapeutic adequacy. Intravenous ascorbate remains a distinct medical intervention requiring separate safety review.

Established uses are strongest for preventing or correcting deficiency and for supporting recognized biochemical functions such as collagen hydroxylation, whereas applications involving fatigue, neuropsychiatric symptoms, microbiome-mediated effects, regenerative medicine, environmental oxidative stress, and supraphysiologic oral dosing remain exploratory or condition-specific.

With appropriate patient selection, evidence-sensitive framing, gastrointestinal tolerability monitoring, renal-risk review, iron-status assessment, hydration planning, de-escalation to maintenance when appropriate, and oncology coordination when relevant, vitamin C can be integrated into individualized clinical nutrition and recovery-oriented care as a context-dependent nutritional cofactor rather than a universal disease-directed therapy. Several proposed clinical applications remain evidence-limited and require confirmation in adequately powered, multicenter clinical trials with predefined patient populations, baseline vitamin C assessment, route-specific exposure definitions, clinically relevant endpoints, and safety monitoring.

## Figures and Tables

**Table 1:** Clinical domains, biological rationale, potential relevance, evidence-sensitive framing, and key cautions for vitamin C.

Clinical Domain	Biological Rationale	Potential Clinical Relevance	Evidence-Sensitive Framing	Key Caution
Orthopedic injury and postoperative recovery	Collagen hydroxylation, extracellular matrix remodeling, oxidative-stress regulation, immune support	Fracture recovery, surgical recovery, soft-tissue healing, postoperative inflammatory stress	Recovery-oriented cofactor	Evidence is insufficient for reliable fracture-union acceleration
Pain and CRPS risk reduction	Oxidative stress, endothelial dysfunction, neurogenic inflammation, tissue repair	CRPS-I risk reduction after fracture or extremity surgery; post-traumatic pain context	Selected CRPS-risk adjunct	Analgesic effect should not be generalized
Regeneration and musculoskeletal repair	Fibroblast function, collagen maturation, angiogenesis, matrix stability	Tendon, ligament, fascia, enthesis, wound, and postoperative tissue repair	Cofactor for repair competence	Independent regenerative effect is unproven
Cancer-supportive care	Redox regulation, nutritional vulnerability, fatigue biology, quality-of-life support	Poor intake, fatigue, treatment-related symptoms, postoperative recovery	Nutritional support; separate oral and IV routes	Do not infer antitumor efficacy
Fatigue and functional recovery	Carnitine synthesis, mitochondrial energy handling, oxidative-stress regulation	Cancer-related fatigue, post-illness fatigue, frailty, inflammatory fatigue	Evaluate within fatigue workup	Fatigue is multifactorial
Neuropsychiatric symptoms	Brain redox balance, catecholamine metabolism, neurotransmitter-related pathways	Low mood, irritability, cognitive complaints, fatigue in nutritionally vulnerable patients	Correct deficiency within psychiatric care	Avoid psychiatric monotherapy framing
Microbiota–gut–brain axis	Intestinal redox balance, microbial metabolites, short-chain fatty acids, vagal and neuroimmune signaling	Mood symptoms, fatigue, cognitive complaints in nutritionally vulnerable patients	Preliminary microbiome-mediated hypothesis	Evidence remains early, population-specific, and non-causal
Skin and wound biology	Dermal collagen synthesis, antioxidant protection, epithelial repair	Bruising, wound healing, postoperative skin recovery, pressure injury	Systemic nutritional cofactor	Do not replace wound or skin management
Vascular and metabolic stress	Nitric oxide bioavailability, endothelial redox balance, vascular collagen	Smoking, diabetes, metabolic syndrome, obesity, environmental oxidative burden	Potential endothelial redox support	Cardiovascular treatment effect is unproven
Environmental toxicant exposure	Antioxidant defense against pollutant-related oxidative stress	Smoking, air pollution, heavy metals, occupational exposure	Redox-oriented nutritional consideration	Do not substitute for exposure control or toxicity treatment

Abbreviations: CRPS, complex regional pain syndrome.

**Table 2:** Patient-selection framework for vitamin C supplementation or selected dose escalation.

Patient Factor	Clinical Examples	Rationale	Clinical Approach	Safety Considerations
Poor intake or nutritional vulnerability	Low fruit/vegetable intake, frailty, alcohol use, restrictive diet, cancer anorexia	Increased risk of insufficiency or deficiency	Nutritional repletion or maintenance supplementation	Assess broader nutrition, protein, B vitamins, vitamin D, magnesium, zinc
Smoking or secondhand smoke	Active smoker, passive smoke exposure	Increased oxidative stress and lower vitamin C status	Consider intake above nonsmoker requirements	Smoking cessation remains primary intervention
Trauma or surgery	Fracture, wound, postoperative recovery, orthopedic surgery	Increased collagen turnover, inflammation, oxidative stress	Ensure adequacy; consider short-term recovery-oriented supplementation	Reliable healing acceleration remains unproven
Orthopedic repair demand	Tendon injury, ligament injury, enthesopathy, regenerative procedures	Collagen and matrix remodeling	Cofactor within repair environment	Combine protein, loading, sleep, rehabilitation
CRPS-risk context	Distal radius fracture, extremity trauma, postoperative pain risk	Oxidative stress and neuroinflammatory mechanisms	Consider use in selected contexts when CRPS risk is clinically relevant	Evidence remains mixed
Cancer or active treatment	Cachexia, poor intake, fatigue, chemotherapy, radiation therapy	Nutritional vulnerability, oxidative stress, symptom burden	Nutritional repletion with oncology coordination	Coordinate with standard therapy and treatment timing
Fatigue, frailty, and neuropsychiatric vulnerability	Post-illness fatigue, cancer fatigue, chronic inflammation, sarcopenia, low mood, cognitive complaints, chronic stress, chronic pain	Carnitine synthesis, redox balance, brain redox physiology, nutritional vulnerability, preliminary microbiota–gut–brain hypotheses	Assess vitamin C status within broader nutritional, medical, psychiatric, sleep, and functional evaluation	Avoid monotherapy framing; evaluate anemia, thyroid dysfunction, sleep disturbance, depression, medications, protein intake, vitamin D, B vitamins, magnesium, and systemic disease
Metabolic and inflammatory vulnerability	Diabetes, obesity, metabolic syndrome, insulin resistance, chronic wounds, poor diet quality	Endothelial stress, oxidative burden, impaired repair, chronic low-grade inflammation	Nutritional assessment in metabolically vulnerable patients	Do not frame supplementation as a primary glucose-lowering or disease-modifying metabolic therapy
Stone or renal-risk patients	Calcium oxalate stones, CKD, hyperoxaluria, dehydration	Potential risk from prolonged high-dose use	Use renal-risk-based dose limitation or monitoring	Check renal function, hydration, urinary risk

Abbreviations: CKD, chronic kidney disease; CRPS, complex regional pain syndrome.

**Table 3:** Route, formulation, and dose-adjustment considerations for clinical vitamin C use.

Category	Examples	Main Clinical Advantage	Main Limitation	Preferred Clinical Use
Oral nutritional dose	Diet, low-dose tablet, multivitamin	Prevents deficiency; easy long-term use	May be insufficient in high-demand states	General maintenance and repletion
Conventional oral supplementation	500 mg^−2^ g/day	Accessible and generally well tolerated	GI symptoms possible in sensitive patients	Smokers, poor intake, modest increased demand
Selected divided oral dose escalation	Divided oral doses adjusted to context	Allows dose adjustment while limiting large boluses	Requires patient education and monitoring	Selected monitored patients with low intake or increased clinical stress
Gastrointestinal tolerance-limited adjustment	Dose reduction when GI symptoms occur	Identifies tolerability boundary	Not a biomarker of systemic requirement	Safety-oriented dose adjustment
Intravenous vitamin C	Sodium ascorbate infusion	Achieves pharmacologic plasma concentrations	Requires medical supervision and safety screening	Monitored medical or investigational contexts
Tablet or capsule	Ascorbic acid or mineral ascorbate tablets	Convenient and standardized	Requires disintegration and dissolution	Maintenance supplementation
Powder	Fine or ultrafine ascorbic acid powder	Flexible dosing; useful for 1–2 g divided dosing	Taste, acidity, requires mixing	Flexible divided dosing
Liquid or dissolved form	Liquid ascorbate or dissolved powder	Useful for swallowing difficulty and rapid ingestion	Stability affected by light, heat, oxygen	Dysphagia, adherence problems, postoperative patients
Buffered or mineral ascorbate	Sodium, calcium, magnesium ascorbate	May improve gastric tolerance	Adds mineral load	Acid-sensitive patients
Sustained-release product	Slow-release vitamin C	May prolong exposure	Less flexible for rapid GI tolerance-limited adjustment	Maintenance rather than rapid titration
Liposomal vitamin C	Phospholipid-encapsulated ascorbate	May improve plasma exposure or tolerance	Product heterogeneity; origin not defined	Selected use when tolerance is limiting
Coarse crystalline form	Crystalline ascorbic acid	Acceptable if fully dissolved	Slower dissolution may reduce usability	Use with sufficient fluid; limit claims to dissolution and tolerability

Abbreviations: GI, gastrointestinal.

## Data Availability

No new data were created or analyzed in this study. Data sharing is not applicable to this article.

## Supplementary Materials

The following supporting information can be downloaded at: https://www.mdpi.com/article/10.3390/nu18142319/s1, Table S1: Representative clinical evidence for vitamin C by clinical domain.
