# Supplementary material for: Vitamin C—Beyond Deficiency: Mechanisms, Clinical Applications, Formulation and Dosing Considerations, and Safety Across Stress-Responsive Conditions
Source: Nutrients. 2026 Jul 15;18(14):2319. doi: 10.3390/nu18142319 (PMC13414856; doi:10.3390/nu18142319)
Supplement: Supplementary file 1 [file nutrients-18-02319-s001.zip › nutrients-4420390-supplementary.pdf]

Supplementary Table S1. Representative clinical evidence for vitamin C by clinical domain.

Clinical domain	Representative evidence	Population or context	Route and dose pattern	Main outcomes	Evidence-sensitive interpretation
Deficiency and nutritional vulnerability	Observational and cross-sectional studies [45–47]	Older adults, hospitalized patients, low-income or nutritionally vulnerable populations	Dietary intake assessment or conventional oral supplementation	Low vitamin C status is associated with frailty, poor intake, hospitalization, and clinical vulnerability	Evidence strongly supports assessment and correction of deficiency or insufficiency in nutritionally vulnerable patients
Tissue repair and wound healing	Systematic review and mechanistic-clinical synthesis [9]	Wound healing, pressure injury, surgical recovery, tissue-repair demand	Mostly oral supplementation; heterogeneous dosing and duration	Potential improvement in selected wound-healing parameters, particularly when baseline status is low or repair demand is high	Adequacy is biologically and clinically defensible, but reliable acceleration of healing in vitamin C-replete patients is not established
Musculoskeletal injury and repair	Systematic and scoping reviews [11,12]	Tendon, ligament, muscle, and musculoskeletal injury contexts	Oral supplementation; variable protocols	Collagen synthesis and oxidative-stress pathways are biologically plausible; clinical outcomes remain heterogeneous	Best framed as a cofactor for repair biology rather than an independent musculoskeletal therapy
Orthopedic trauma and CRPS risk	Randomized trials and meta-analyses [22–24]	Distal radius fracture, extremity trauma, postoperative or post-traumatic CRPS-risk contexts	Commonly oral vitamin C 500 mg/day for approximately 45–50 days	Some studies report reduced CRPS-I incidence, whereas others show less consistent effects	Cautious consideration is reasonable in selected CRPS-risk contexts; analgesic or generalized orthopedic benefit should not be inferred
Cancer-supportive care	Systematic reviews and pilot randomized evidence [27–30]	Cancer patients, supportive care, treatment-related symptoms, hematologic oncology or	Oral nutritional supplementation or pharmacologic intravenous ascorbate;	Quality-of-life, fatigue, nutritional status, plasma vitamin C correction, and supportive endpoints; survival benefit remains	Oral and IV approaches must be separated; use should remain nutritional, supportive, investigational, or pharmacologic depending on

		transplantation settings	route-dependent exposure	unproven	route and context
Fatigue and functional recovery	Randomized trial and systematic review [31,32]	Office workers, cancer-related fatigue, post-viral or chronic fatigue contexts	Mainly intravenous vitamin C in selected studies; heterogeneous protocols	Some studies suggest symptom improvement, but baseline vitamin C status is often unmeasured	Fatigue is multifactorial; vitamin C should be evaluated as one nutritional variable, especially when low status or systemic illness is suspected
Neuropsychiatric symptoms	Systematic review and meta-analysis [18,33]	Low mood, depressive symptoms, cognitive complaints, medically ill or nutritionally vulnerable populations	Oral supplementation in heterogeneous clinical trials	Some improvement in mood-related outcomes, particularly in low-status or vulnerable groups	Evidence supports correction of deficiency or insufficiency within standard care, not psychiatric monotherapy
Microbiota–gut–brain axis	Pilot, randomized, and mechanistic microbiome studies [34–38]	Healthy or young adults with suboptimal vitamin C status; gut microbiome and neuroimmune signaling contexts	Oral supplementation; variable dose and duration	Changes in gut microbial composition and mental vitality-related outcomes have been reported in selected populations	Preliminary and hypothesis-generating; causal neuropsychiatric benefit is not established
Vascular and metabolic vulnerability	Systematic reviews, umbrella reviews, and meta-analysis [40–44]	Endothelial dysfunction, hypertension, metabolic syndrome, type 2 diabetes, oxidative-stress contexts	Oral supplementation; variable dose, duration, and co-supplementation	Effects on endothelial measures, blood pressure, glycemic, or lipid outcomes are heterogeneous	Supports potential redox-oriented nutritional consideration in higher-risk groups; disease-modifying cardiovascular or metabolic claims are not justified
Formulation and pharmacokinetics	Pharmacokinetic studies and formulation reviews [5,6,48,61–66]	Healthy adults, smokers, formulation-comparison settings	Oral tablets, powders, sustained-release, liposomal preparations, and IV ascorbate	Oral absorption is saturable; IV achieves pharmacologic plasma concentrations; formulation claims vary by product	Route and formulation should be reported explicitly; improved exposure or tolerability does not necessarily imply superior clinical efficacy

Safety and monitoring	Safety reviews, renal-risk studies, G6PD reports, laboratory-interference studies [21,56–60,69–73]	Stone-prone patients, CKD, oncology, critical illness, G6PD deficiency, IV ascorbate exposure	Conventional oral, prolonged high-dose oral, or high-dose IV exposure	GI intolerance, oxalate-related renal risk, iron overload context, hemolysis risk with IV in G6PD deficiency, and glucose-meter interference	Safety review should be proportional to route, dose, duration, renal risk, hydration, iron status, oncology context, and IV-specific risks

Abbreviations: CKD, chronic kidney disease; CRPS, complex regional pain syndrome; GI, gastrointestinal; G6PD, glucose-6-phosphate dehydrogenase; IV, intravenous. This table provides an evidence-sensitive narrative summary and should not be interpreted as formal GRADE assessment.
